# Supplementary material for: Environmentally Relevant Lead Exposure Alters Cell Morphology and Expression of Neural Hallmarks During SH-SY5Y Neuronal Differentiation
Source: bioRxiv. 2025 Feb 21:2025.02.17.638689. Preprint. [Version 1] doi: 10.1101/2025.02.17.638689 (PMC11870460; doi:10.1101/2025.02.17.638689)
Supplement: Supplement 1 [file media-1.pdf]

- Images
- Metadata
- Names and Types
- Groups
- Correct Illumination Apply

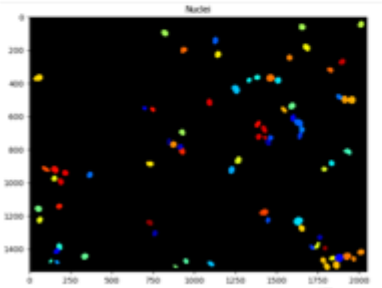

- **Identify Primary Objects**
- **Identify Primary Objects**

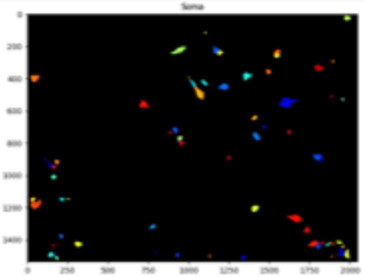

- Enhance or Suppress Features
- **Identify Secondary Objects**

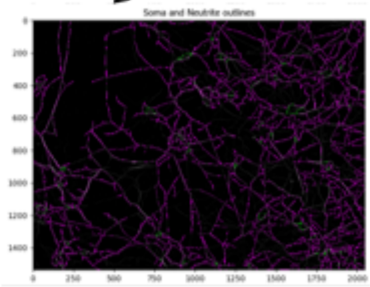

- Convert Objects To Image
- **Morphological Skeleton**

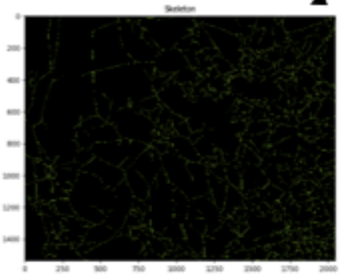

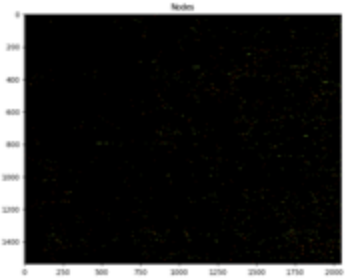

- Measure Image Skeleton
- Overlay Outlines
- Overlay Outlines
- Save Images
- Save Images
- Export to Database
- Export to Spreadsheet
