# Supplementary material for: Environmentally Relevant Lead Exposure Alters Cell Morphology and Expression of Neural Hallmarks During SH-SY5Y Neuronal Differentiation
Source: bioRxiv. 2025 Feb 21:2025.02.17.638689. Preprint. [Version 1] doi: 10.1101/2025.02.17.638689 (PMC11870460; doi:10.1101/2025.02.17.638689)
Supplement: Supplement 2 [file media-2.pdf]

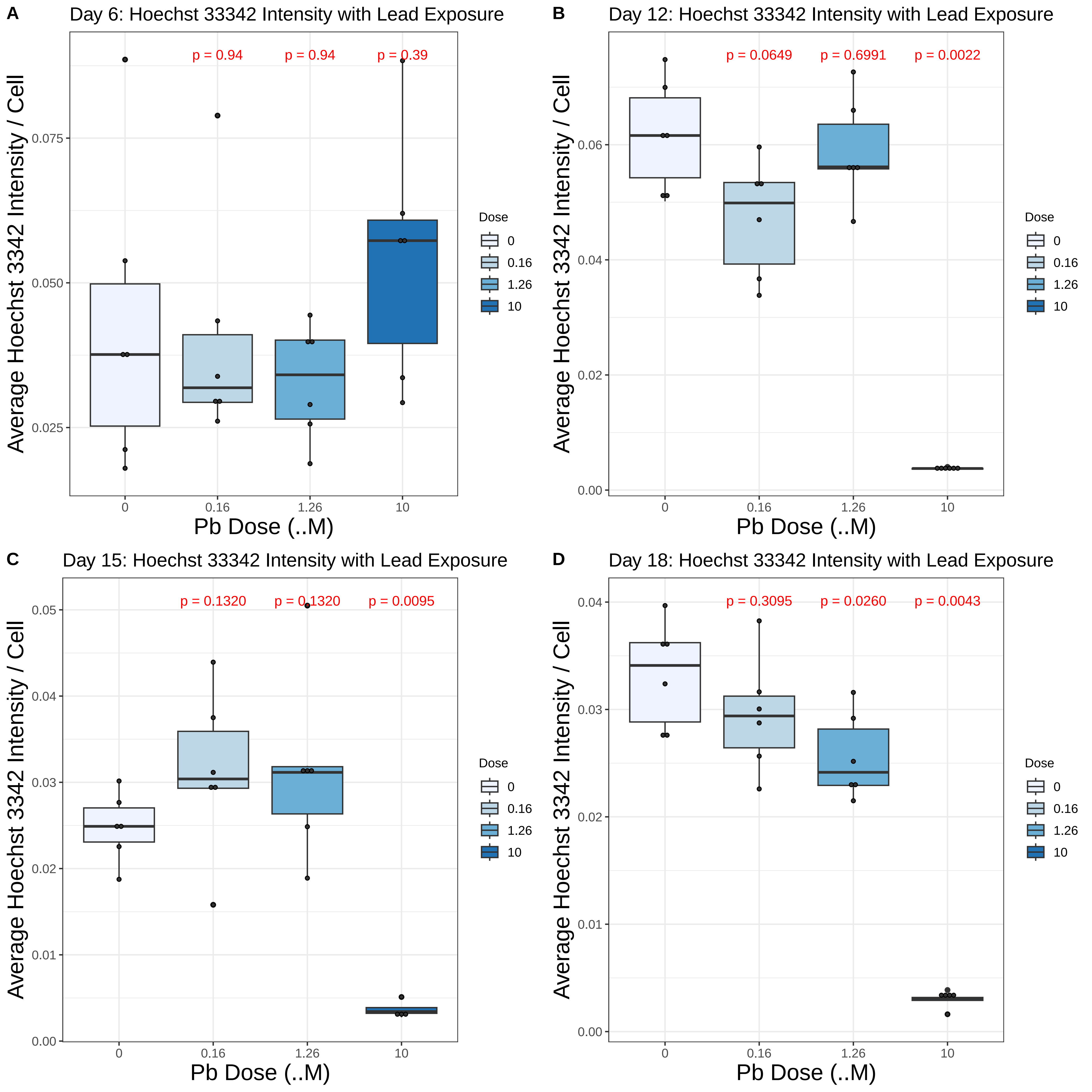
A
Day 6: Hoechst 33342 Intensity with Lead Exposure
p = 0.94
p = 0.94
p = 0.39
Average Hoechst 3342 Intensity / Cell
0.075
0.050
0.025
0
0.16
1.26
10
Pb Dose (..M)
Dose
0
0.16
1.26
10
B
Day 12: Hoechst 33342 Intensity with Lead Exposure
p = 0.0649
p = 0.6991
p = 0.0022
Average Hoechst 3342 Intensity / Cell
0.06
0.04
0.02
0.00
0
0.16
1.26
10
Pb Dose (..M)
Dose
0
0.16
1.26
10
C
Day 15: Hoechst 33342 Intensity with Lead Exposure
p = 0.1320
p = 0.1320
p = 0.0095
Average Hoechst 3342 Intensity / Cell
0.05
0.04
0.03
0.02
0.01
0.00
0
0.16
1.26
10
Pb Dose (..M)
Dose
0
0.16
1.26
10
D
Day 18: Hoechst 33342 Intensity with Lead Exposure
p = 0.3095
p = 0.0260
p = 0.0043
Average Hoechst 3342 Intensity / Cell
0.04
0.03
0.02
0.01
0.00
0
0.16
1.26
10
Pb Dose (..M)
Dose
0
0.16
1.26
10
