# Supplementary material for: Environmentally Relevant Lead Exposure Alters Cell Morphology and Expression of Neural Hallmarks During SH-SY5Y Neuronal Differentiation
Source: bioRxiv. 2025 Feb 21:2025.02.17.638689. Preprint. [Version 1] doi: 10.1101/2025.02.17.638689 (PMC11870460; doi:10.1101/2025.02.17.638689)
Supplement: Supplement 3 [file media-3.docx]

**SH-SY5Y Staining Protocol**

Materials Needed:

| - Cell line in culture: SH-SY5Y | - Antibodies/stains of interest: |
| --- | --- |
| - Media used for cells | - - β tubulin III conjugate (Thermo, 50-4510-82)     - eFluor 660, host: mouse |
| - 16% paraformaldehyde (PFA) | - - GAP43 Primary (Abcam, ab75810)     - Rabbit monoclonal |
| - PBS (pH 7.4) | - - GAP43 Secondary (Abcam, ab150077)     - Fluor 488, host: goat, anti-rabbit |
| - Normal Goat Serum (NGS) | - - Hoechst 33342 (Thermo, H3570) |
| - Tin foil/dark space to incubate |  |

Protocol:

1. Make PBS-T – you’ll need enough to rinse each well 10x
   1. Add Tween 20 to PBS at a 1:1000 concentration.
      1. 50mL PBS + 50µL Tween 20
2. Fix Cells with PFA – prepare and use in **chemical hood**
3. Dilute 16% to a 4% PFA solution (1:4) in media.
4. Gently remove media from cells.
5. Add enough 4% PFA solution to cover the bottom of each well (e.g., 100µL for a 24 well plate).
6. Incubate at room temperature for 20 minutes.
7. Remove 4% PFA solution and rinse each well with PBS-T one time.
8. Permeabilize cells with Triton X – prepare and use in **biosafety cabinet**
9. Add Triton X to PBS at a 1:1000 dilution.
10. Add enough Triton X-PBS solution to cover the bottom of each well.
11. Incubate at room temperature for 15 minutes.
12. Remove Triton X-PBS and rinse each well with PBS-T two times.
13. Primary Stain – work in the dark/protect antibodies from light as much as possible, prepare and use in **biosafety cabinet**
14. Create primary stain solution:
    1. For 1 24-well plate, 100µL per well:
       1. 2.5mL NGS
       2. 1.25µL Hoechst 33342 (0.5µL/mL)
       3. 16.5µL β-tubulin III conjugate antibody (6.6µL/mL)
       4. 12.5µL GAP43 primary (5µL/mL)
15. Add enough primary stain solution to cover the bottom of each well.
16. Incubate 1 hour in the dark at room temperature.
17. Remove primary stain and rinse with PBS-T three times.
18. Secondary Stain – work in the dark/protect antibodies from light as much as possible, prepare and use in **biosafety cabinet**
19. Create secondary stain solution:
    1. For 1 24-well plate, 100µL per well:
       1. 2.5mL NGS
       2. 7.5µL GAP43 secondary (3µL/mL)
20. Add enough primary stain solution to cover the bottom of each well.
21. Incubate 1 hour in the dark at room temperature.
22. Remove primary stain and rinse with PBS-T three times.
23. Add enough PBS-T back to each well to cover.
24. Imaging
25. Image on CV8000 using the following channels:
    1. 405 nm (Hoechst)
    2. 488 nm (GAP43)
    3. 660 nm (β-tubulin III)
