# Supplementary figures and images for: Environmentally Relevant Lead Exposure Alters Cell Morphology and Expression of Neural Hallmarks During SH-SY5Y Neuronal Differentiation
Source: bioRxiv. 2025 Feb 21:2025.02.17.638689. Preprint. [Version 1] doi: 10.1101/2025.02.17.638689 (PMC11870460; doi:10.1101/2025.02.17.638689)

Nuclei

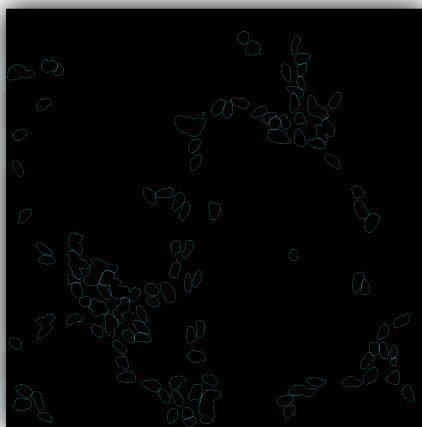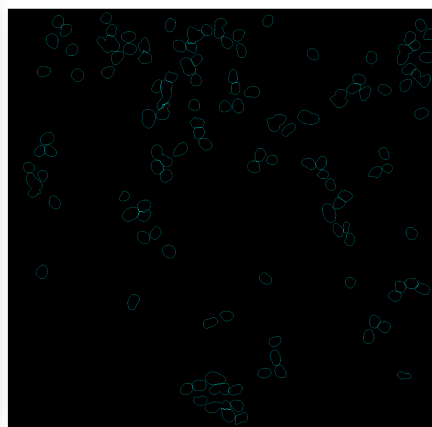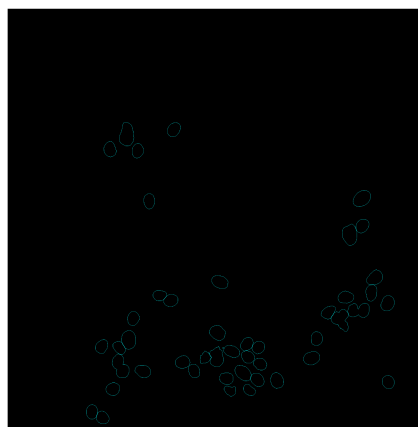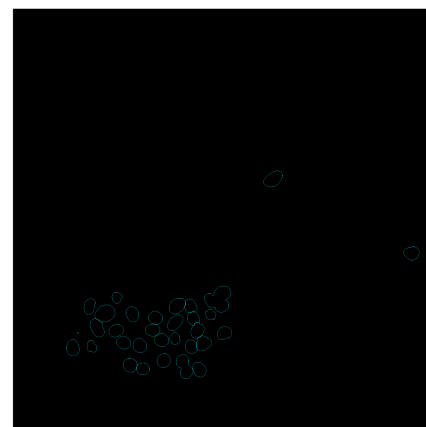

Soma

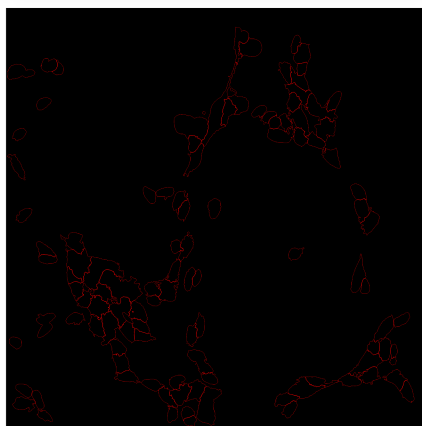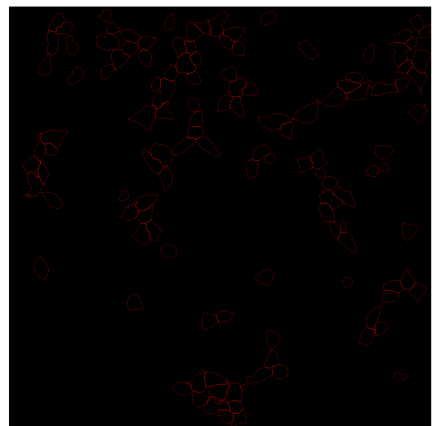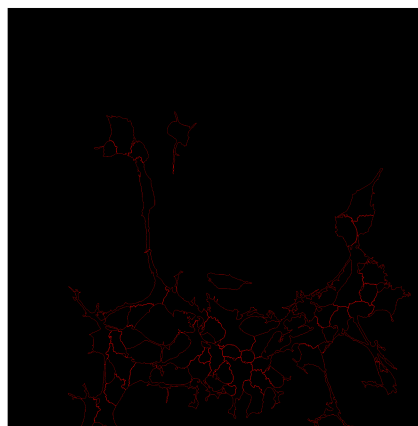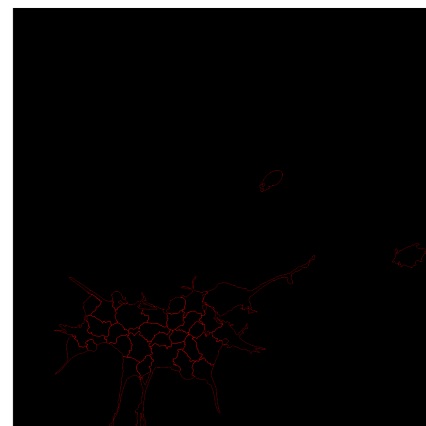

Neurite Outline

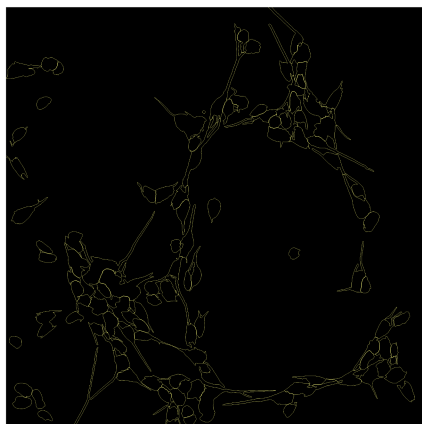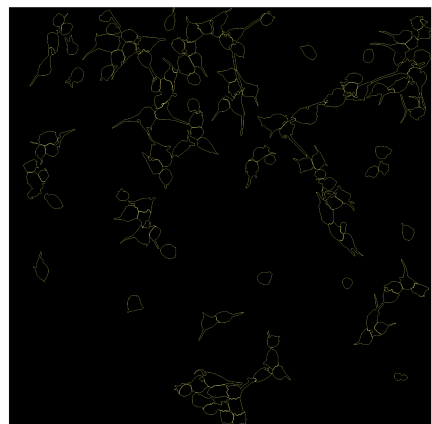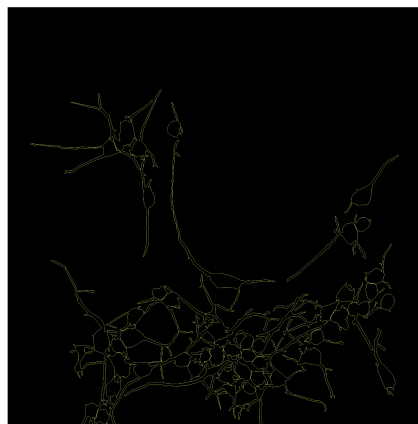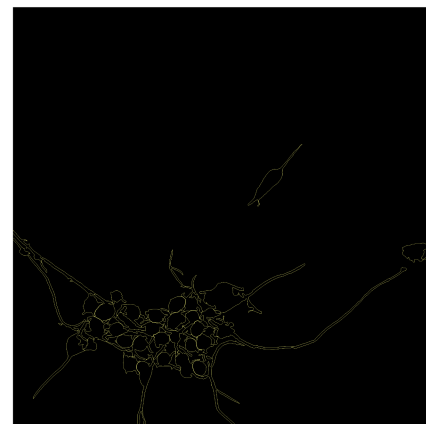

Neurite Skeleton

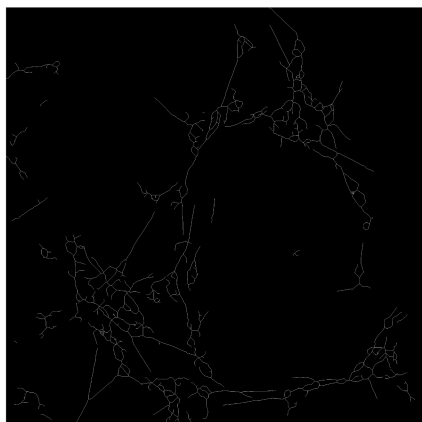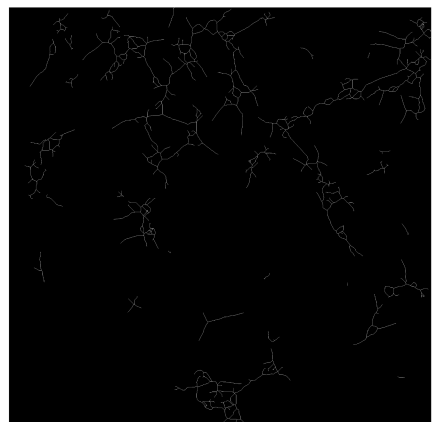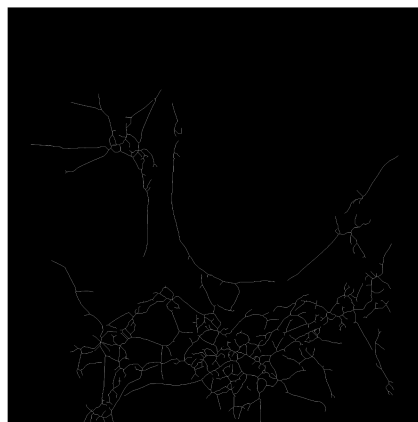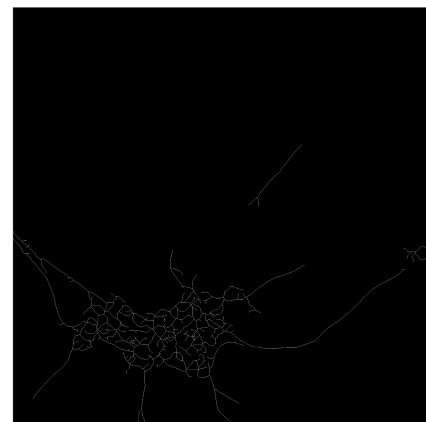

Day 6

Day 12

Day 15

Day 18

Supplement: Supplement 7 [file media-7.pdf]
